# Advances in multimodal imaging for adrenal gland disorders: integrating CT, MRI, and nuclear medicine

**DOI:** 10.1007/s11604-025-01732-6

**Published:** 2025-01-11

**Authors:** Kota Yokoyama, Mitsuru Matsuki, Takanori Isozaki, Kimiteru Ito, Tomoki Imokawa, Akane Ozawa, Koichiro Kimura, Junichi Tsuchiya, Ukihide Tateishi

**Affiliations:** 1 Department of Diagnostic Radiology, Institute of Science Tokyo, Bunkyo-ku, Tokyo, Japan; 2 Department of Pediatric Medical Imaging, Jichi Children’s Medical Center Tochigi, Jichi Medical University, Shimotsuke, Tochigi, Japan; 3 Department of Radiology, School of Medicine, Jichi Medical University, Shimotsuke, Tochigi, Japan; 4 Department of Radiology, National Cancer Center, Tokyo, Japan

**Keywords:** Adrenal gland, Multimodal imaging, Computed tomography, Magnetic resonance imaging, Nuclear medicine, Functional imaging

## Abstract

Adrenal diseases pose significant diagnostic challenges due to the wide range of neoplastic and non-neoplastic pathologies. Radiologists have a crucial role in diagnosing and managing these conditions by, leveraging advanced imaging techniques. This review discusses the vital role of computed tomography (CT), magnetic resonance imaging (MRI), and nuclear medicine in adrenal imaging, and focuses on morphological and functional evaluations. First, the anatomy and physiology of the adrenal glands are described, followed by a discussion on ectopic adrenocortical adenomas and how they develop. The concepts and imaging findings of congenital diseases, such as congenital adrenal hyperplasia (CAH), adrenal rest tumors, and adrenocortical nodular disease, considering recent updates to the WHO Classification of Tumours (5th ed.) terminology are highlighted. The diagnostic value of dynamic contrast-enhanced CT and chemical-shift MRI for identifying adrenocortical adenomas are emphasized, alongside the use of adrenocortical scintigraphy such as ^131^I-adosterol scintigraphy for diagnosing Cushing’s disease, Cushing’s syndrome (CS), subclinical CS, and ectopic adrenocorticotropic hormone-producing tumors. Systemic complications associated with CS, and the diagnosis and treatment of pheochromocytomas, paragangliomas (PPGLs), and neuroblastomas, will also be discussed focusing on ^123^I-metaiodobenzylguanidine (MIBG) imaging and ^131^I-MIBG therapy. Pitfalls in ^123^I-MIBG imaging and the increasing importance of diagnosing hereditary PPGLs due to increased genetic testing are also be discussed. Additionally, the broad differential diagnosis for adrenal masses—including malignancies like adrenal carcinoma, metastases, and malignant lymphoma, as well as benign conditions like myelolipoma and ganglioneuroma, and complications, such as adrenal hemorrhage, infarction, and infections—will be outlined. The goal of this review was to provide an overview of adrenal diseases that includes the most recent information for radiologists to stay updated on the latest imaging techniques and advancements that can ensure accurate diagnosis and effective management.

## Introduction

Adrenal gland disorders pose significant diagnostic challenges for radiologists because of their diverse pathologies. The WHO classification identifies 18 distinct tumors, including 10 adrenal cortical tumors and 8 adrenal medulla and extra-adrenal paraganglia tumors [1], which requires radiologists to have a comprehensive understanding of adrenal pathology and anatomy for accurate diagnosis and management. Imaging, particularly computed tomography (CT), magnetic resonance imaging (MRI), and nuclear medicine, is essential for evaluating adrenal disorders by assessing function, staging, metastasis, and guiding treatment. Dynamic contrast-enhanced CT [2, 3] and chemical-shift imaging (CSI) on MRI [4, 5] have improved the diagnosis of adrenocortical adenomas. Functional imaging has traditionally had a crucial role in diagnosing conditions, such as Cushing’s syndrome (CS), Cushing's disease (CD), and primary aldosteronism (PA), and its importance has increased with advances in nuclear medicine treatments for pheochromocytomas, paragangliomas (PPGLs), and neuroblastomas [6–10]. Additionally, the increasing frequency of diagnosing hereditary PPGLs highlights the growing importance of correlating genetic mutations with imaging findings [11–13]. Recent reports have also linked adrenal complications to immune checkpoint inhibitors (ICIs) [14], adding more complexity to the diagnosis.

Despite these advancements, the broad differential diagnoses of adrenal lesions require not only mastery of diagnostic techniques but also a deeper understanding of each condition. This review discusses the adrenal anatomy, major diseases, and rare but important conditions that radiologists must be familiar with as well as key imaging techniques and advancements critical for their accurate diagnosis and effective management.

## Anatomy, development, and physiology of the adrenal glands

### Anatomy and abnormalities

**Fig. 1:**
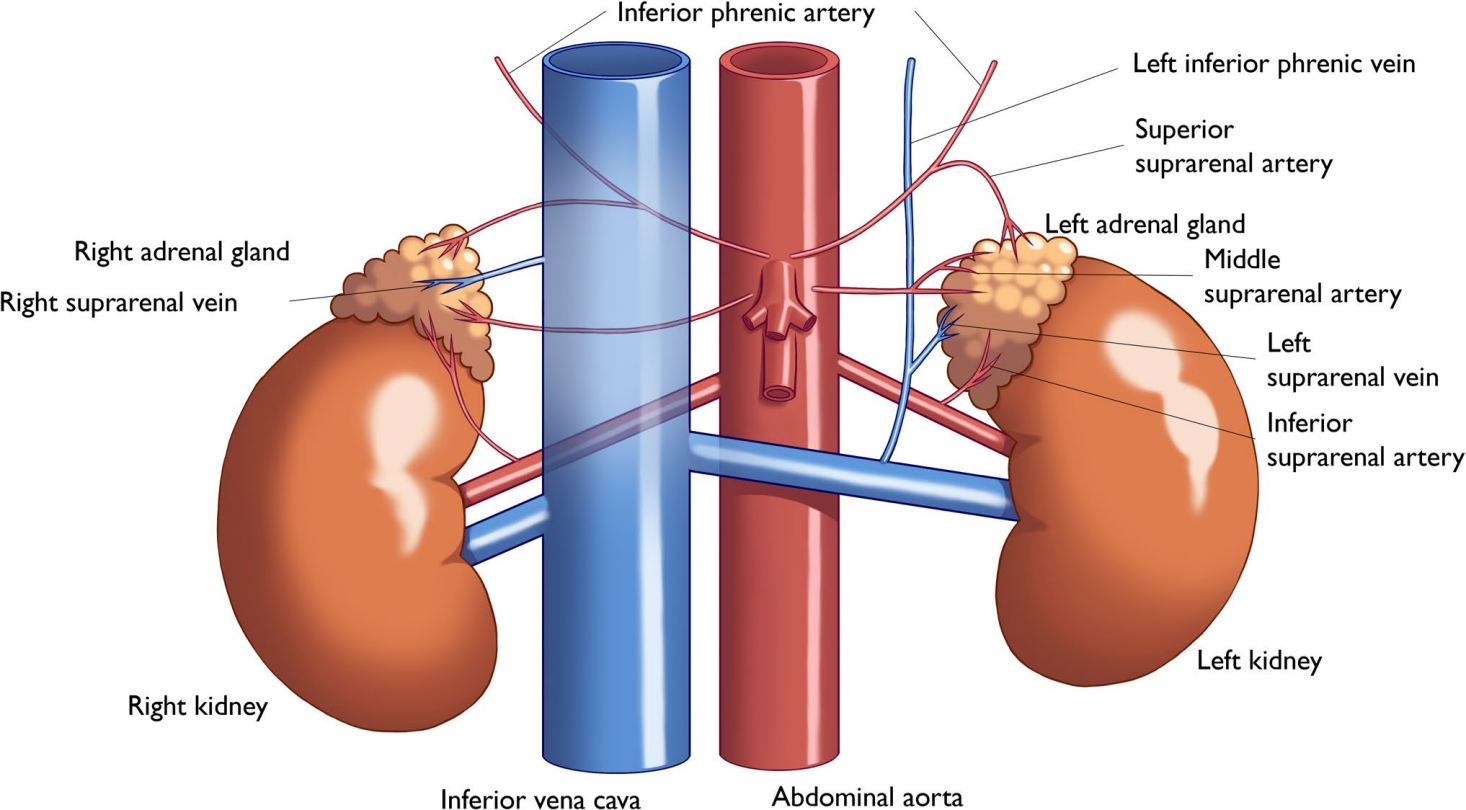
Anatomy of the adrenal glands and surrounding structures. This illustration shows the anatomical relationships of the adrenal glands to the nearby organs and vasculature. The key components include the right-adrenal gland and vein, inferior vena cava, abdominal aorta, and left kidney. The adrenal glands’ arterial blood supply is provided by the superior, middle, and inferior suprarenal arteries, and the glands are drained by the left- and right-suprarenal veins. This figure highlights the location of the left- and right-adrenal glands and the vascular connections to both kidneys. Illustration by Akane Ozawa

**Fig. 2:**
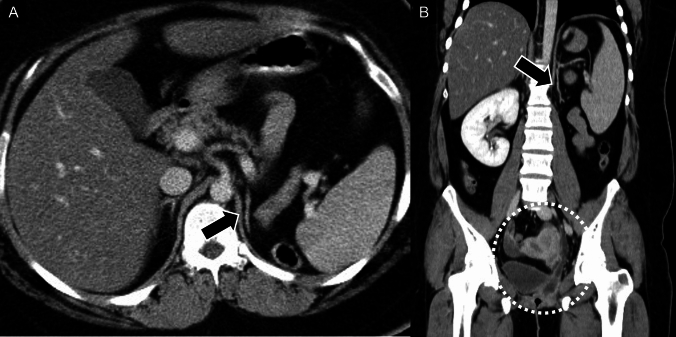
Discoid adrenal gland in a patient with Herlyn–Werner–Wunderlich syndrome. Computed tomography imaging in a 20-year-old female presenting with amenorrhea showing a bicornuate uterus with a cystic structure continuous, with the cervix on the left side of the pelvic floor (**B**: white dotted circle), along with agenesis of the left kidney. The left-adrenal gland is flattened and elongated, displaying the characteristic discoid shape (**A**, **B**: black arrow), which is commonly associated with unilateral renal agenesis

Adrenal glands are small, triangular organs located above the kidneys in the retroperitoneal space. Each gland weighs approximately 5–6 g [15] and measures approximately 5 cm in length [15, 16]. Each adrenal gland is supplied by three adrenal arteries and are drained by a single adrenal vein (Fig. 1). On imaging, they typically appear as an inverted Y shape on CT or MRI axial or coronal images [16], and the right- and left-adrenal glands’ maximum widths are approximately 6.1 mm and 7.9 mm, respectively [17], and their cranio-caudal lengths are < 4 cm [18]. In newborns, the adrenal glands are relatively large, being about one-third the size of the kidneys, and they gradually shrink during infancy [19, 20]. Variations in adrenal size may depend on the intrauterine environment and stress factors [21]. Abnormalities such as ‘pancake’ adrenal occur in various conditions, including renal agenesis or ectopic kidney [22]. “Pancake” adrenal also is referred to as “discoid,” “straight,” “elongated,” and “lying down adrenal” (Fig. 2). After nephrectomy, it is important to recognize that structures with such morphology represent deformed adrenal glands. Horseshoe adrenal gland, a rare congenital anomaly, involves fusion across the midline and is sometimes referred to as a “butterfly” adrenal gland [23]. This condition is often associated with other congenital anomalies, such as asplenia, neural tube defects, Cornelia de Lange syndrome, and certain renal abnormalities [23, 24]. Imaging is critical for identifying this condition and related midline defects.

### Adrenal cortex development and ectopic adrenal cortical adenomas

**Fig. 3:**
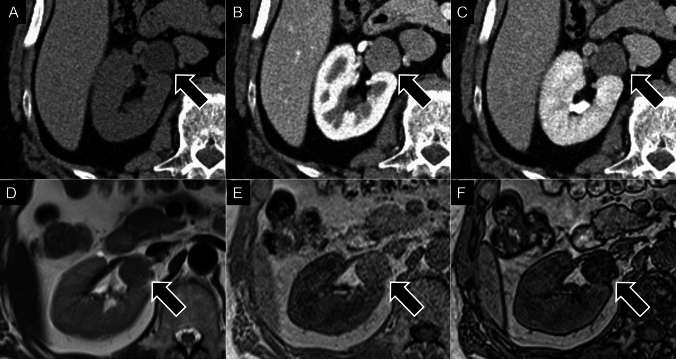
Ectopic adrenocortical adenoma. A 60-year-old male was found to have a mass near the right-renal hilum during a health screening. On unenhanced computed tomography, the mass demonstrates low attenuation with a measurement of 8 Hounsfield Units (**A**: black arrow), while contrast-enhanced images reveal a washout tendency (**B**, **C**: black arrow). On the T2-weighted image, the mass shows slightly low intensity (**D**: black arrow). On chemical-shift imaging, the lesion exhibits high signal intensity on in-phase images (**E**: black arrow) and signal loss on opposed-phase images (**F**: black arrow), indicating the presence of intracytoplasmic fat. The mass was surgically resected and histopathologically diagnosed as an ectopic adrenocortical adenoma

The adrenal cortex originates from the urogenital ridge, which also forms the kidneys and reproductive organs [25]. Ectopic adrenal cortical adenomas can develop in various locations due to the aberrant migration of adrenal cells during embryogenesis, such as near the renal hilum (Fig. 3) or within the broad ligament of the uterus [26].

### Functional anatomy of the adrenal glands and hyperplasia

**Fig. 4:**
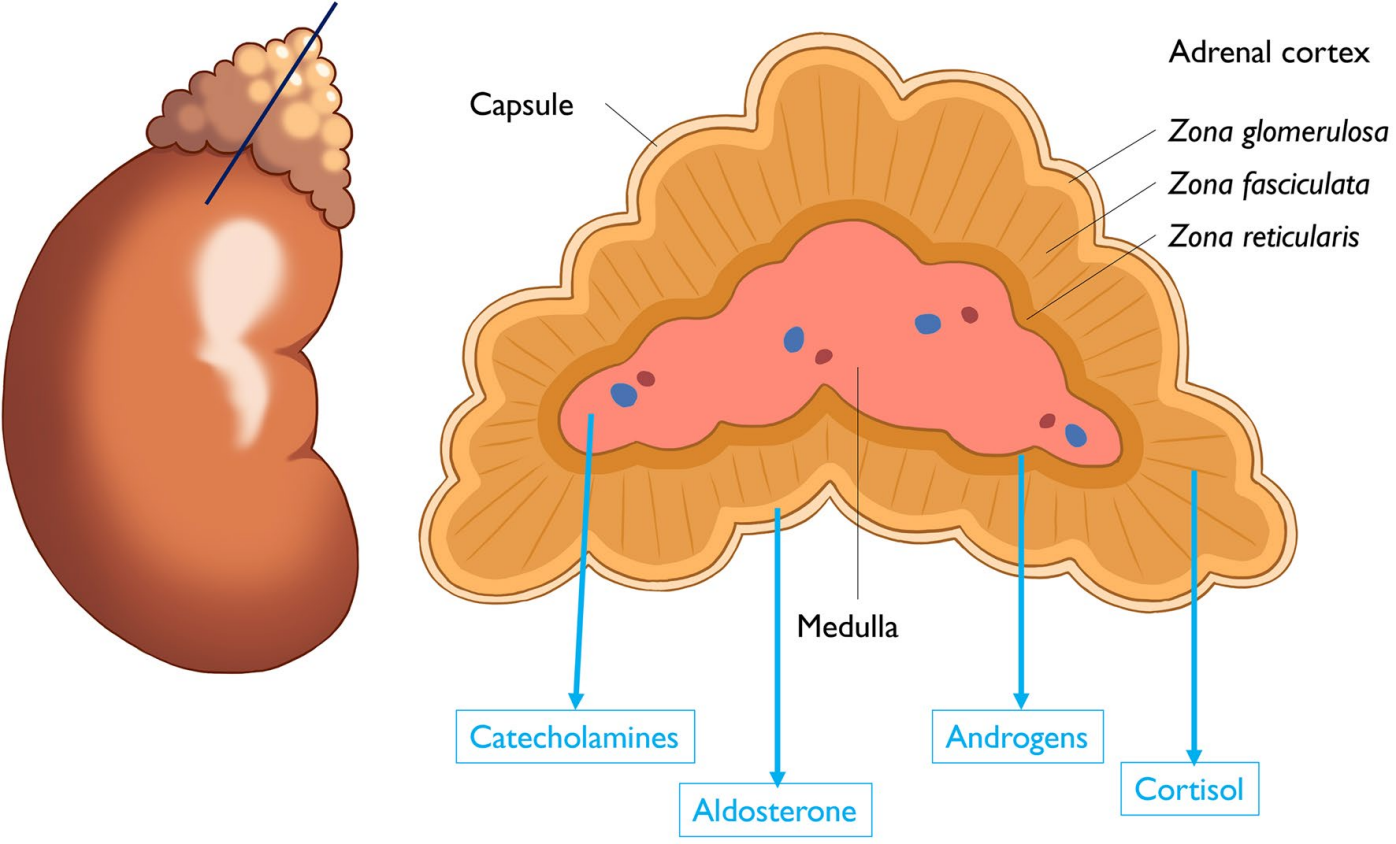
Functional anatomy of the adrenal cortex and medulla. This illustration shows the zonal structure of the adrenal cortex, which is divided into three layers: the zona glomerulosa, which produces aldosterone; the zona fasciculata, responsible for cortisol production; and the zona reticularis, which secretes androgens. The adrenal medulla is shown as the inner region, which produces catecholamines. Illustration by Akane Ozawa

The adrenal glands have two distinct parts: the cortex and medulla. The cortex produces steroid hormones critical for physiological functions and is divided into three zones (Fig. 4):

- *Zona glomerulosa*: Produces mineralocorticoids (e.g., aldosterone) that regulate the fluid and electrolyte balance.
- *Zona fasciculata*: Produces glucocorticoids (e.g., cortisol), which are critical for glucose and energy metabolism and are regulated by the hypothalamic–pituitary–adrenal (HPA) axis.
- *Zona reticularis*: Produces adrenal androgens, such as dehydroepiandrosterone (DHEA) and its sulfate (DHEA-S), which are involved in sexual development.

**Fig. 5:**
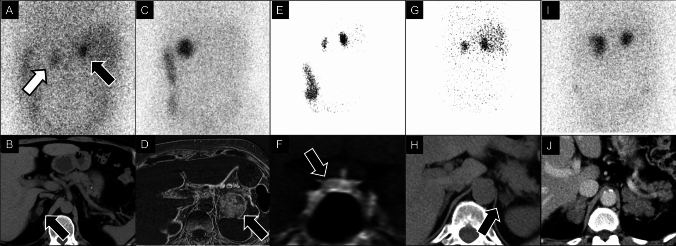
Posterior to anterior view of ^131^I-adosterol scintigraphy and related imaging findings under various conditions. (**A**) and (**B**): Subclinical Cushing syndrome in a 50-year-old female. Adrenocortical scintigraphy showing moderate radiotracer uptake in the right-adrenal mass (**A**: black arrow) with mild contralateral adrenal suppression (**A**: white arrow), suggesting subclinical Cushing’s syndrome. Non-contrast computed tomography (CT) showing a hypo-attenuating mass on right-adrenal gland consistent with adenoma (B: black arrow). (**C**) and (**D**): Cushing syndrome in a 60-year-old female. Adrenocortical scintigraphy showing marked radiotracer uptake in the adenoma with obvious contralateral suppression, indicative of Cushing syndrome (**C**). Chemical-shift imaging subtraction image highlights the fat content in the adenoma (**D**: black arrow). (**E**) and (**F**): ACTH-dependent Cushing’s disease in a 42-year-old female. Bilateral adrenal uptake is detected by adrenocortical scintigraphy (**E**), and pituitary magnetic resonance imaging reveals a hypovascular adenoma on delayed contrast-enhanced imaging (**F**: black arrow). **G** and **H**: Bilateral micronodular adrenocortical disease in a 67-year-old female. Although initially suspected as a unilateral functional adrenal adenoma by CT (**H**), adrenocortical scintigraphy showed symmetrical high uptake bilaterally, leading to the diagnosis of bilateral micronodular adrenocortical disease, preventing surgery (**G**). **I** and **J**: Bilateral macronodular adrenocortical disease in a 71-year-old male. Adrenocortical scintigraphy showing high uptake bilaterally (**I**) with multiple nodules on contrast-enhanced CT (**J**), leading to the diagnosis of bilateral macronodular adrenocortical disease.

Understanding anatomy and feedback mechanisms is crucial for diagnosing and managing CS, CD, PA, and similar conditions as well as adrenocorticotropic hormone (ACTH)-producing tumors. Long-term steroid use may lead to adrenal atrophy, obscuring underlying conditions. Adrenocortical scintigraphy is important for evaluating adrenal conditions (Fig. 5).

The medulla, derived from the neural crest, functions in the sympathetic nervous system by secreting catecholamines (e.g., epinephrine, norepinephrine) in response to stress and regulating blood pressure, heart rate, and metabolism. Metabolic increases during stress are visible in 2-deoxy-2-[^18^F]fluoroglucose (^18^F-FDG) positron-emission tomography (PET)/CT. Understanding this anatomy is key to diagnosing PPGLs and neuroblastoma and to interpreting ^123^I- MIBG (MIBG) scintigraphy and nuclear medicine treatments that use ^131^I-MIBG.

Overall, the adrenal glands are critical for homeostasis, regulating fluid balance, stress response, metabolism, and sexual development [27]. Although small in size, the adrenal glands have an important effect on various bodily functions and are essential to overall health. Understanding the structure and function of adrenal glands is key to interpreting imaging and diagnosing adrenal disorders.

## Adrenal cortex lesion

### Hyperplasia

#### Congenital adrenal hyperplasia (CAH)

Congenital adrenal hyperplasia (CAH) is a genetic disorder primarily caused by 21-hydroxylase deficiency, which impairs cortisol and aldosterone production [28]. This deficiency often results in adrenal hyperplasia due to the overproduction of adrenal androgens, primarily DHEA and androstenedione, under the influence of elevated adrenocorticotropic hormone ACTH levels. In its classic form, CAH can present early, often with serious complications, such as salt-wasting crises, which can be life-threatening, and ambiguous genitalia in females. It is characterized by a complete or near-complete deficiency of 21-hydroxylase activity. Conversely, non-classic CAH typically presents later and tends to be milder. It is caused by a partial deficiency of 21-hydroxylase activity, resulting in less severe hormonal imbalances [29].

Ultrasonography is particularly useful in neonates and infants, with key findings including limb length > 20 mm, mean width > 4 mm, and normal corticomedullary differentiation, strongly suggesting CAH [30–32]. Lobulated or cerebriform surfaces and stippled echogenicity are highly sensitive and specific [32]. CT can reveal adrenal enlargement, often caused by persistent ACTH stimulation, while MRI offers superior soft-tissue contrast resolution, enabling a more detailed assessment of adrenal morphology without radiation, allowing safe, repeated evaluations in pediatric patients. Additionally, ^123^I-MIBG scintigraphy can help differentiate adrenal hyperplasia from other adrenal lesions when the conventional imaging is inconclusive [33] and is useful for detecting testicular adrenal rest tumors (TART) [34].

**Fig. 6:**
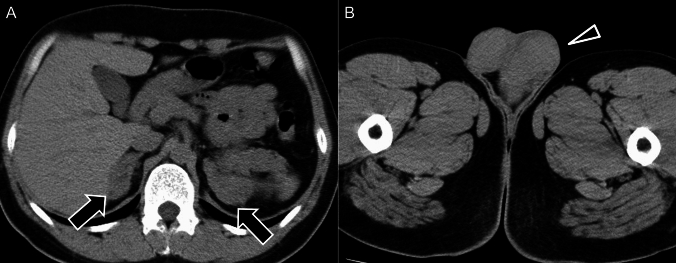
Testicular adrenal rest tumors in congenital adrenal hyperplasia. (**A**–**B**) Unenhanced computed tomography (CT) in an 18-year-old male showing multinodular masses in both adrenal glands (arrows) and soft-tissue masses in both testes (arrowhead), consistent with adrenal rest tumors. These findings are typical of testicular adrenal rest tumors associated with congenital adrenal hyperplasia

TARTs are benign lesions composed of adrenal cortical tissue that can develop in the testes of male patients with CAH. They resemble Leydig cell tumors and are often bilateral are usually small, multiple, and detectable by ultrasonography, especially in post-pubertal males (Fig. 6) [35].

Early and accurate imaging is crucial for guiding treatment and preventing complications in CAH. It is managed with early and continuous cortisol replacement to prevent adrenal crises and normalize hormone levels [36]. Timely imaging reduces morbidity and mortality in classic CA and helps physicians manage symptoms and monitor outcomes in non-classic CAH [29].

#### Cushing’s disease (CD)

CD is a form of CS caused by an ACTH-producing pituitary adenoma, now referred to as a pituitary neuroendocrine tumor (PitNET) [37]. CD typically presents in adults between 20 and 50 years of age but can also occur in children, where it is often associated with growth retardation [38]. Pituitary-driven excess ACTH leads to bilateral adrenal hyperplasia, which can be detected by CT, MRI, or adrenocortical scintigraphy (Fig. 5C). Pituitary MRI is crucial for identifying PitNET, often a microadenoma < 10 mm. Up to 50% of microadenomas are too small to detect by standard MRI due to their size. Dynamic contrast-enhanced imaging [39] and thin-slice protocols [40] aid in detecting microadenomas [41]. The recommended protocol has been discussed elsewhere [41]. Additionally, 3-T MRI has proven to be highly effective in providing a detailed evaluation of both normal pituitary anatomy [42] and microadenomas [41]. If the MRI results are inconclusive, bilateral inferior petrosal sinus sampling (BIPSS) confirms the pituitary source of ACTH, with high sensitivity and specificity for ACTH-dependent CD [43–45].

The primary treatment for CD is the surgical removal of the PitNET, typically via transsphenoidal surgery. If surgery is not successful, additional options include radiation therapy or medical management [46]. Although imaging is key in the diagnosis of CD, clinical information and biochemical testing remain essential for confirming the diagnosis and guiding treatment.

#### Ectopic ACTH syndrome (EAS)

Ectopic ACTH-producing tumors, which cause CS, are most often associated with malignancies located outside the pituitary gland. Common sources include the lungs, particularly in cases of small-cell lung carcinoma, bronchial, and thymic carcinoids [47–49]. Other ectopic sources include medullary thyroid carcinoma, pancreatic neuroendocrine tumors (NET), pheochromocytomas, and, less commonly, gastrointestinal carcinoids [47–49]. EAS typically presents with severe hypercortisolism, including central obesity, purple striae, hypertension, glucose intolerance, osteoporosis, and avascular femoral head necrosis [50]. Steroid-induced myopathy, which causes proximal muscle weakness, severely impairs mobility and quality of life [51]. Excessive cortisol also increases the risk of opportunistic infections, such as *Pneumocystis jirovecii* pneumonia [51]. Diagnosing EAS is challenging because the ACTH source is often hard to identify. Imaging studies, including CT and MRI, are typically employed to locate the tumor. Functional imaging, such as octreotide scintigraphy, detects NETs expressing somatostatin receptors [50]. In recent years, ^68^Ga-DOTA^0^-Tyr^3^-octreotate (DOTATATE) PET/CT has shown promise for identifying these tumors with high sensitivity [52, 53], although its availability is limited in certain regions, including Japan. BIPSS is crucial for distinguishing between pituitary and ectopic ACTH sources [45]. Non-invasive tests, such as the desmopressin stimulation test, also show promise in differentiating CD from EAS [54].

Managing EAS requires a multidisciplinary approach, focusing on identifying and resecting the primary tumor when possible [55]. When the tumor cannot be resected or remains unidentified, medical therapies, such as ketoconazole or metyrapone [56], are used to control cortisol production. The management of complications, such as steroid-induced myopathy, and prevention of opportunistic infections are crucial for improving patient outcomes.

### Adrenal cortex neoplasm

#### Adrenocortical nodular disease

Adrenocortical nodular disease refers to a group of benign nodular proliferations within the adrenal cortex. Historically, these conditions have been described as “adrenal cortical nodular hyperplasia” or “micronodular and macronodular adrenocortical hyperplasia.” Although ACTH-independent macronodular adrenal hyperplasia was once a familiar term, it is now considered outdated [57]. The latest WHO classification includes sporadic nodular adrenocortical disease and bilateral micronodular adrenocortical disease (BMACD), which encompasses primary pigmented nodular adrenocortical disease (PPNAD) and isolated micronodular adrenocortical disease (i-MAD), replacing ACTH-independent macronodular adrenal hyperplasia (AIMAH) [57].

#### Adrenal cortical adenomas: Cushing’s syndrome and primary aldosteronism

Adrenal cortical adenomas are common benign tumors that are often incidentally discovered during imaging, and lipid-rich adenomas comprise approximately 70% of these. They show unenhanced CT attenuation of ≤ 10 HU, which is highly specific for adenomas, since other adrenal tumors rarely present with such low attenuation [58]. CSI reliably detects small amounts of fat and distinguishes adenomas from non-adenomas. Recent meta-analyses have highlighted the high sensitivity (94%) and specificity (95%) of CSI for diagnosing adenomas, confirming its diagnostic accuracy [5]. For differentiation, CSI findings should be interpreted by assessing signal loss on opposed-phase images relative to in-phase images, which is characteristic of adenomas due to their intracytoplasmic lipid content. This interpretation is crucial for distinguishing lipid-rich adenomas from non-adenomas. DWI and apparent diffusion coefficient (ADC) maps have also been assessed for differentiating adrenal masses, but their utility is limited due to overlapping ADC values between adenomas, myelolipomas, and carcinomas [59].

**Fig. 7:**
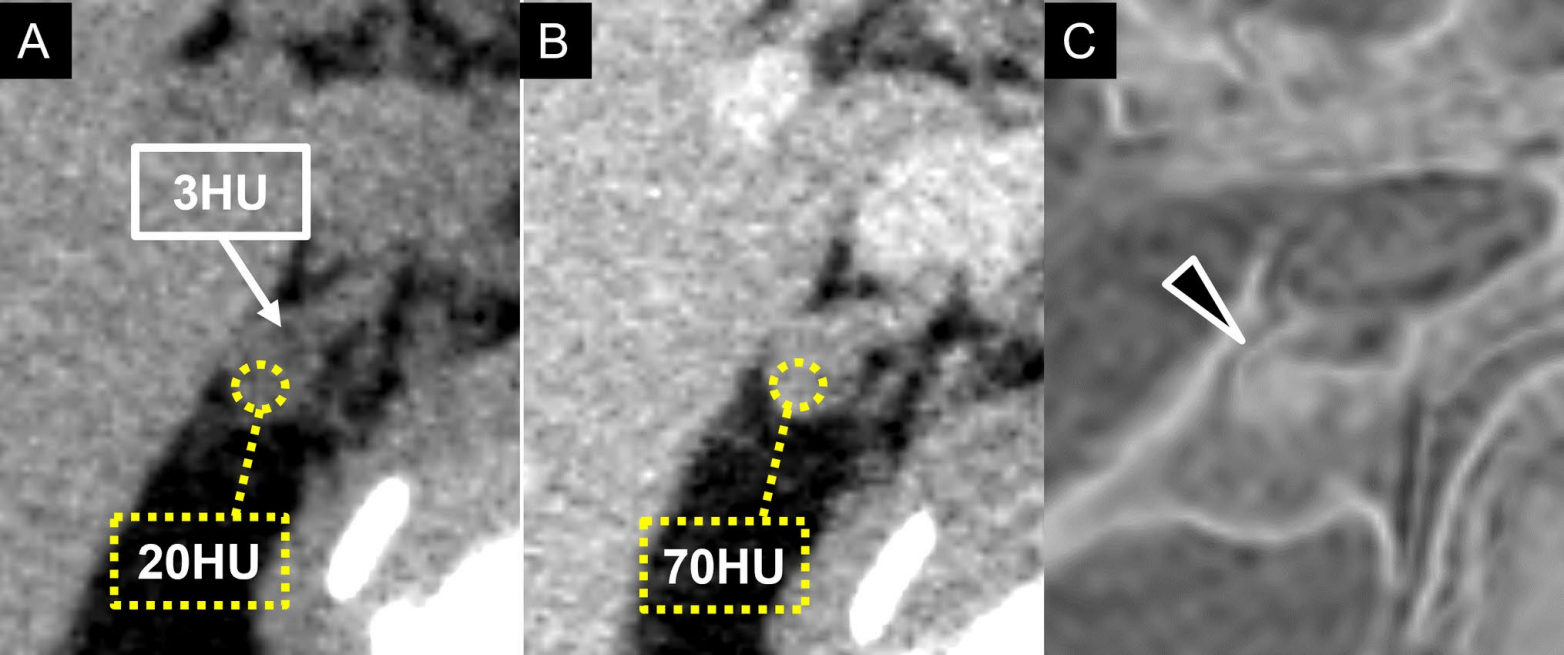
Computed tomography and magnetic resonance imaging findings in an aldosterone-producing adenoma. A 73-year-old male with hypertension and nocturia diagnosed with primary aldosteronism based on an aldosterone–renin ratio > 200 in the captopril challenge test. **A** Unenhanced CT shows a low-attenuation area (3 HU) anteriorly in the right-adrenal mass (arrow), indicating the presence of fat content. **B** The posterior portion of the lesion demonstrates increased attenuation from 20 HU on the unenhanced phase to 70 HU on the portal venous phase (with a relative enhancement ratio of 250%), exceeding the cutoff value of 210%, consistent with a cortical adenoma. **C** A subtraction image of chemical-shift imaging suggests lipid content within the lesion. Adrenal venous sampling confirmed this region as an aldosterone-producing adenoma, and the patient underwent surgical resection for definitive treatment

Differentiating lipid-poor adenomas from non-adenomas, such as metastases, is crucial. Traditionally, differentiation relied on calculating the absolute and relative washout rates from contrast-enhanced CT [3]. Adenomas typically exhibit rapid contrast washout, with an absolute washout rate > 60% and a relative washout rate > 40%, but these methods require delayed imaging at 15 min after injecting contrast, which can be challenging in routine clinical practice. In response to this challenge, the Relative Enhancement Ratio (RER) has been proposed as a more practical and efficient alternative [2], using only precontrast and portal venous-phase imaging (Fig. 7). The RER is calculated using the following formula: {(contrast-enhanced attenuation [HU] minus unenhanced attenuation [HU]) divided by unenhanced attenuation [HU]} × 100%. This method, with a threshold of RER > 210%, has shown 86% sensitivity and 95% specificity for diagnosing lipid-poor adenomas, providing a non-invasive efficient option suited for modern workflows.

Once an adenoma is diagnosed, determining its functional status is crucial, especially for CS and primary aldosteronism (PA). Functional adenomas can produce excess cortisol, leading to CS or excess aldosterone, leading to PA. For CS, guidelines recommend biochemical tests such as the 1-mg overnight dexamethasone suppression test, urinary free cortisol, and late-night salivary cortisol for initial screening [60, 61]. Adrenocortical scintigraphy is not routinely recommended in the current guidelines but remains highly valuable for its functional assessment [62–64], and this technique is helpful in diagnosing CS, characterized by marked radiotracer uptake and contralateral suppression due to negative feedback from elevated ACTH (Fig. 5C, D). This presentation differs from CD, which shows bilateral marked uptake due to elevated ACTH secretion from a pituitary adenoma (Fig. 5E, F). Subclinical CS (SCS) generally shows low radiotracer uptake and contralateral suppression (Fig. 5A, B), aiding in differentiation from overt CS [65]. This differentiation is important because distinctly different management strategies are required. CS often requires surgical removal of the adenoma, whereas SCS may be managed conservatively depending on the symptoms and overall patient health.

For PA, diagnostic evaluation includes measuring the plasma aldosterone concentration and plasma renin activity, with an elevated aldosterone–renin ratio suggesting PA [66, 67]. Confirmatory tests, such as the saline infusion test or captopril challenge test, verify autonomous aldosterone secretion. Adrenal venous sampling (AVS), the gold standard for distinguishing unilateral from bilateral disease, provides critical insights for management, while CT imaging complements AVS by identifying structural abnormalities [66]. Adrenocortical scintigraphy is particularly useful when AVS and CT are inconclusive [68]. In PA, no abnormal adrenal accumulation is typically observed. However, functional adenomas can be visualized using dexamethasone suppression, which suppresses ACTH production and physiological radiotracer uptake, enabling the detection of functioning adenomas [69]. Surgery is recommended for unilateral cases, while mineralocorticoid receptor antagonists are used for bilateral cases [67]. Appropriate diagnosis and treatment can reduce long-term health risks.

In summary, imaging techniques, such as unenhanced CT and chemical-shift MRI, are critical for diagnosing adrenal adenomas. The RER offers a useful alternative if the lipid content is low, and although DWI and ADC maps have been explored, they have limited diagnostic value. For functional assessment, combining biochemical testing with imaging tools like AVS and adrenocortical scintigraphy ensures comprehensive evaluation and tailored management of these entities.

#### Adrenal cortical carcinoma

**Fig. 8:**
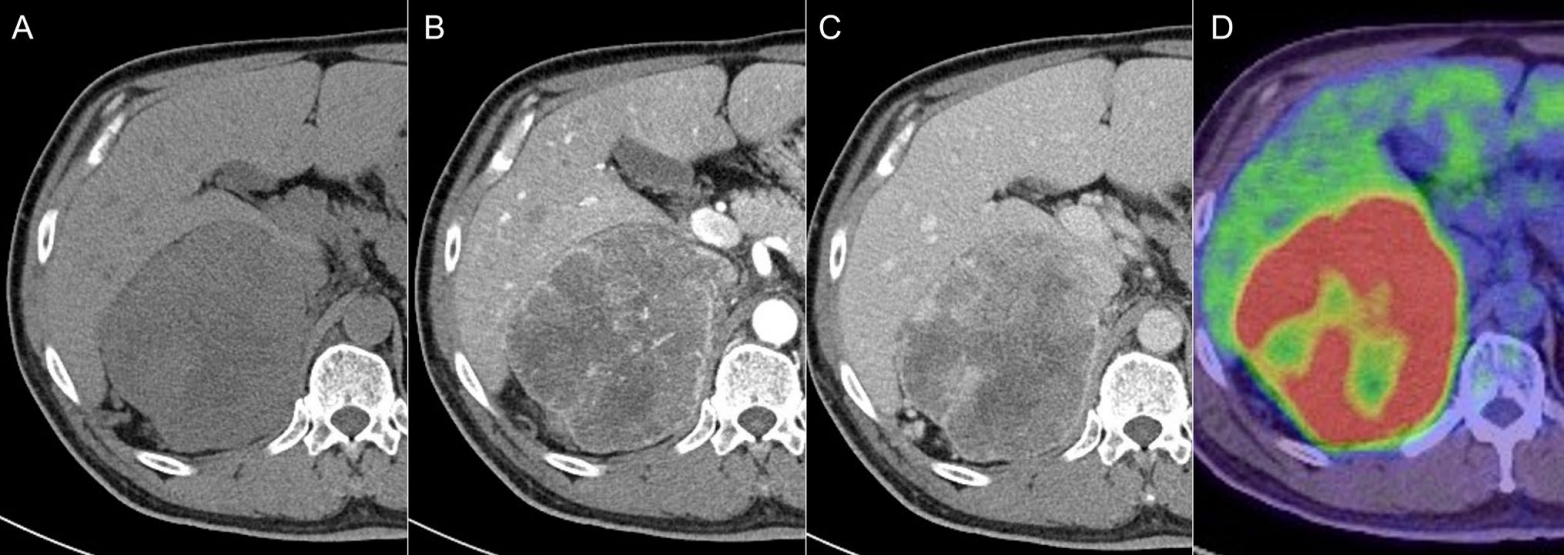
Imaging findings of adrenocortical carcinoma. **A** Unenhanced computed tomography (CT) showing a 10-cm right-adrenal mass with a heterogeneous low density. **B** and **C** Enhanced CT images showing fine tumor vessels and heterogeneous gradual enhancement in the mass. **D** (^18^F)-fluorodeoxyglucose positron-emission tomography/computed tomography showing intense accumulation in the lesion with a maximum standardized uptake value of 19.27, indicating high metabolic activity consistent with adrenocortical carcinoma

Adrenal cortical carcinoma (ACC) is a rare highly malignant tumor of the adrenal cortex that typically appears as a large irregular mass with heterogeneous enhancement, necrosis, and hemorrhage, often exceeding 6 cm (Fig. 8) [70–72]. Calcifications are also commonly encountered [71, 72]. The masses show delayed contrast washout of < 40%, which distinguishes them from benign adenomas that usually show rapid washout [2]. ACCs tend to aggressively invade surrounding structures, such as the renal vein, inferior vena cava (IVC), and liver, with invasion often visible on imaging. MRI is particularly useful for detailed evaluation of IVC [73] and hepatic invasion [74], and CSI helps detect the lipid content typical of adenomas but not of ACCs [75, 76]. ^18^F-FDG PET/CT shows higher uptake in ACCs than in adenomas, and studies have highlighted its good diagnostic performance [77]. ACC may be discovered incidentally, especially when hormonally inactive, but symptoms related to hormone excess often lead to its diagnosis when active. Pathologically, ACC is characterized by increased mitotic activity, atypical mitotic figures, and necrosis, with the Weiss [78] or Helsinki score [79, 80] used to differentiate between benign and malignant tumors. A study found a significant correlation (77%, *p* < 0.0001) between ^18^F-FDG uptake and Weiss scores, underscoring the role of ^18^F-FDG PET/CT in evaluating malignancy [81].

The prognosis is poor, with a 5-year survival rate between 10 and 65% depending on the stage at diagnosis and surgical success [82]. Complete surgical excision is the primary treatment, often followed by adjuvant therapies, such as mitotane and radiation. Early and accurate diagnosis based on imaging is critical for improving outcomes [83].

## Adrenal medulla and extra-adrenal paraganglia tumors

### Pheochromocytomas and paragangliomas (PPGLs)

**Fig. 9:**
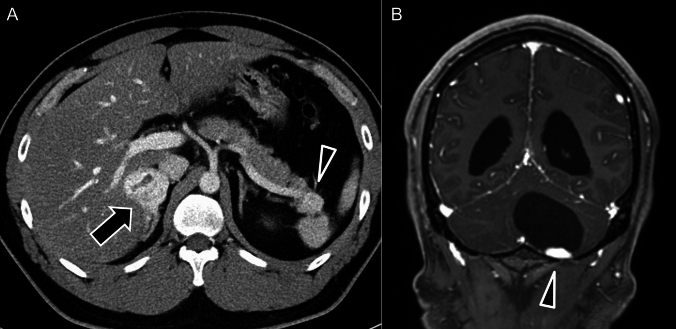
Pheochromocytoma related to von Hippel–Lindau disease. **A** Enhanced computed tomography showing a homogeneously enhancing mass in the right-adrenal gland (black arrow), consistent with pheochromocytoma, and a mass in the tail of the pancreas (arrowhead), suggestive of a pancreatic neuroendocrine tumor. **B** Gadolinium-enhanced T1-weighted magnetic resonance imaging (gadolinium-T1-weighted imaging) showing a cystic tumor with an intensely enhancing mural nodule (arrowhead) in the left cerebellum, consistent with a hemangioblastoma. These findings suggest von Hippel–Lindau disease with the characteristic involvement of pheochromocytoma, pancreatic neuroendocrine tumor, and cerebellar hemangioblastoma.

**Fig. 10:**
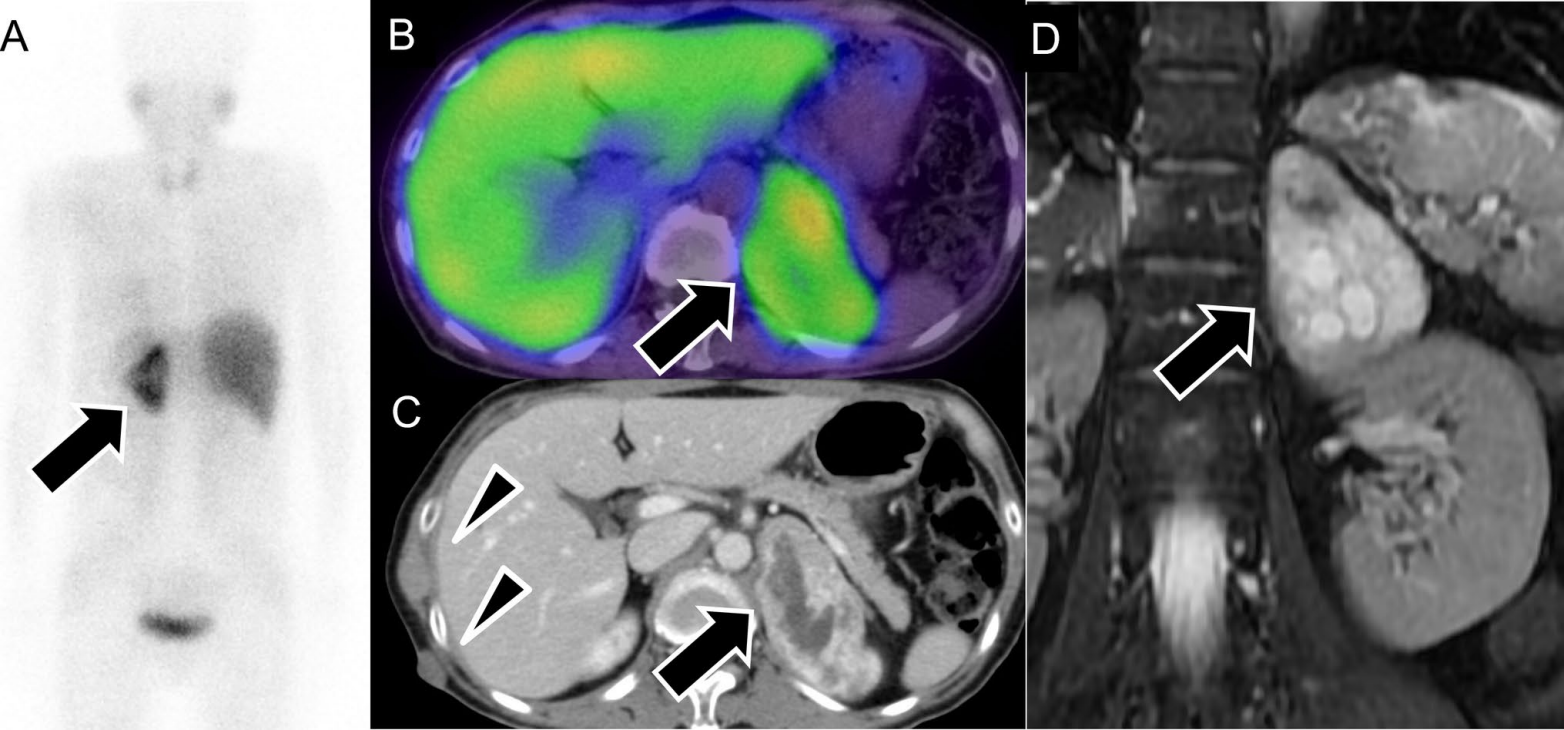
Pheochromocytoma associated with neurofibromatosis type 1. **A** ^18^F-fluorodeoxyglucose positron-emission tomography/computed tomography (^18^F-FDG PET/CT) maximum-intensity projection shows increased ^18^F-FDG uptake in the left-adrenal gland (black arrow). **B** ^18^FDG PET/CT fusion image showing intense ^18^F-FDG accumulation in the left-adrenal pheochromocytoma (black arrow). **C** Contrast-enhanced CT revealing a hypervascular cystic mass in the left-adrenal gland (black arrow), consistent with pheochromocytoma, and a right-sided subcutaneous nodule (arrowheads) in the chest wall, consistent with neurofibromatosis. **D** Coronal T2-weighted imaging shows the characteristic “light bulb” sign, indicating a pheochromocytoma in the left-adrenal gland (black arrow)

**Fig. 11:**
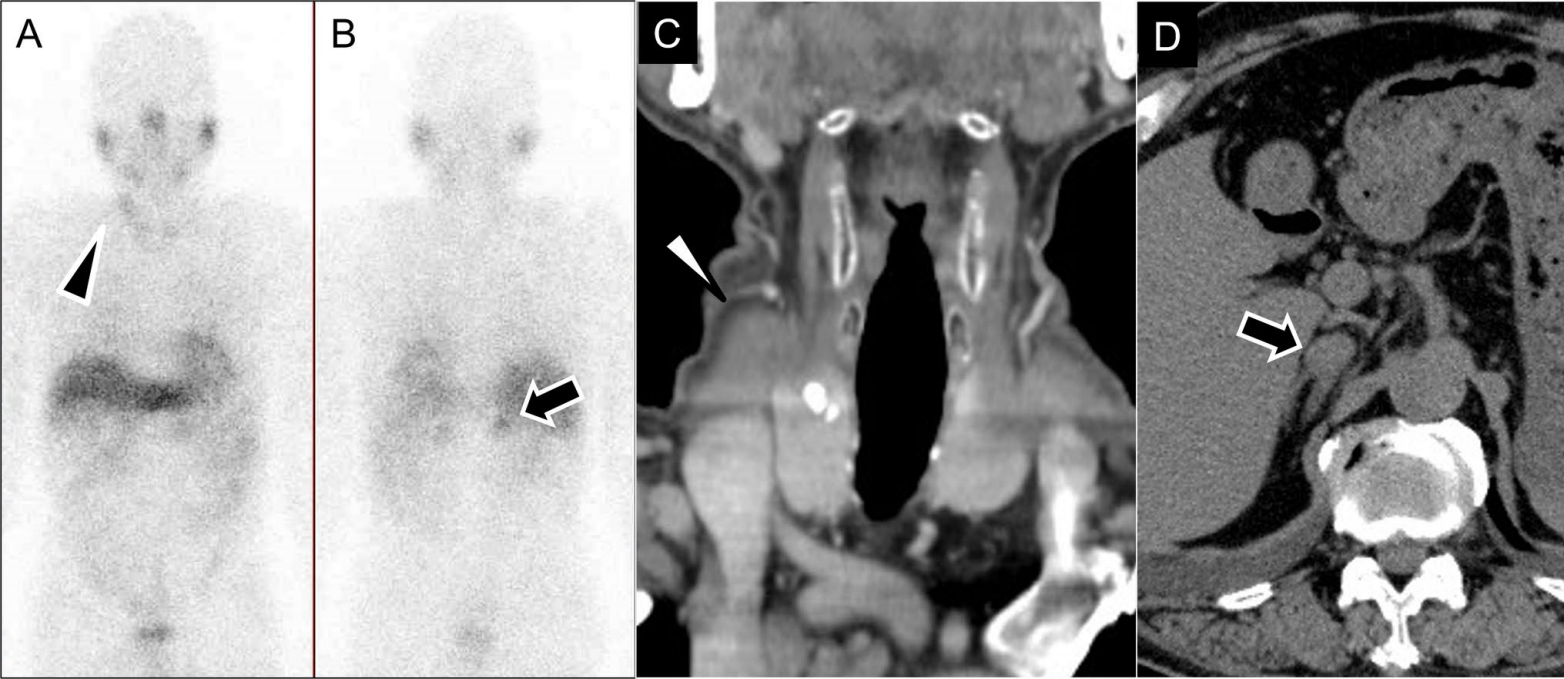
Pheochromocytoma in multiple endocrine neoplasia type 2A. **A**, **B** ^123^I-metaiodobenzylguanidine scintigraphy images showing significant radiotracer accumulation in the right thyroid (arrowhead) and left-adrenal gland (arrow), consistent with medullary thyroid cancer and pheochromocytoma, respectively. **C** Enhanced computed tomography (CT) of the neck showing a low-density area with coarse calcifications (arrowhead) at the upper pole of the right lobe of the thyroid gland. Total thyroidectomy confirmed the diagnosis of medullary thyroid cancer. **D** Unenhanced CT of the abdomen showing a left-adrenal nodule (arrow), pathologically confirmed as pheochromocytoma

**Fig. 12:**
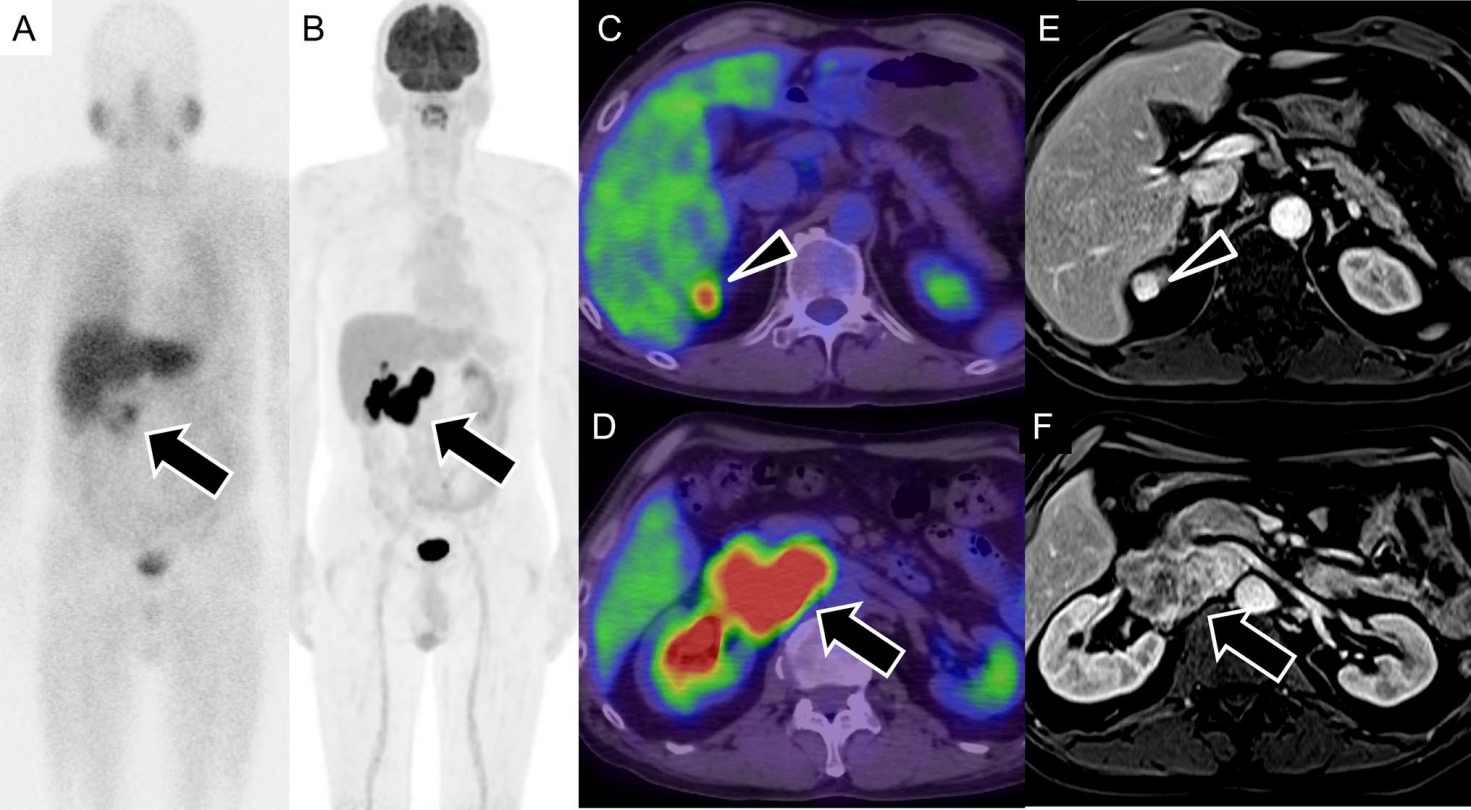
Pheochromocytoma in the PGL4 syndrome (SDHB mutation). A 50-year-old man with confirmed succinate dehydrogenase subunit B (SDHB) gene mutation. **A** ^123^I-metaiodobenzylguanidine (^123^I-MIBG) scintigraphy showing radiotracer uptake in the retroperitoneal tumor (arrow), a characteristic finding in pheochromocytoma; however, most of the tumor showing minimal uptake. **B** ^18^F-fluorodeoxyglucose positron-emission tomography/computed tomography (^18^FDG PET/CT) demonstrates intense ^18^FDG uptake in the lesion’s border, which is more prominent than ^123^I-MIBG uptake (arrow), highlighting the typical discrepancy between low MIBG uptake and high ^18^FDG accumulation in SDHB-associated tumors. **C** and **D** ^18^FDG PET/CT fusion images confirm the high ^18^FDG uptake in the retroperitoneal tumor (arrow), with additional uptake in a right-renal mass (arrowhead). **E** and **F** Contrast-enhanced magnetic resonance imaging in the early phase revealing hypervascularity in both the right-renal mass, pathologically confirmed as clear-cell carcinoma (arrowhead), and the right-adrenal heterogeneous mass, confirmed as pheochromocytoma (arrow). In PGL4 syndrome, associated conditions, such as pituitary adenoma, gastrointestinal stromal tumor, and renal-cell carcinoma, can be found alongside pheochromocytoma.

PPGLs are rare NETs that originate from chromaffin cells. Pheochromocytomas typically arise in the adrenal medulla, whereas paragangliomas develop in extra-adrenal paraganglia. These tumors secrete catecholamines, causing episodic hypertension, palpitations, headaches, and sweating. With advancements in imaging and genetic screening, many PPGLs are now detected incidentally, particularly in patients with hereditary syndromes. PPGLs are frequently associated with hereditary syndromes driven by key genetic mutations in the Von Hippel–Lindau (VHL), neurofibromatosis type 1 (NF1), rearranged during transfection (RET), and succinate dehydrogenase B (SDHB) genes. These mutations correspond to specific syndromes, including VHL syndrome (Fig. 9), NF1 (Fig. 10), multiple endocrine neoplasia type 2 (Fig. 11), and pheochromocytoma/paraganglioma syndrome type 4 (Fig. 12), respectively [84].

On CT, PPGLs often appear as highly vascular masses, showing intense enhancement during the arterial phase (Fig. 9 A). On MRI, PPGLs appear as well-defined, hyperintense masses on T2-weighted imaging (T2WI), often showing the characteristic “lightbulb” sign (Fig. 10D) [85, 86]. T1WI shows a “salt-and-pepper” appearance [86–88], indicating microhemorrhages and high vascular flow. For succinate dehydrogenase (SDH)-related paraganglioma, especially those with SDHB mutations, ADC values on MRI can serve as a useful biomarker. These tumors typically show lower ADC values than those of non-SDH-mutated tumors, which aids in differentiation and management decisions and reflects their more clinically aggressive behavior and poorer prognosis [89, 90].

**Fig. 13:**
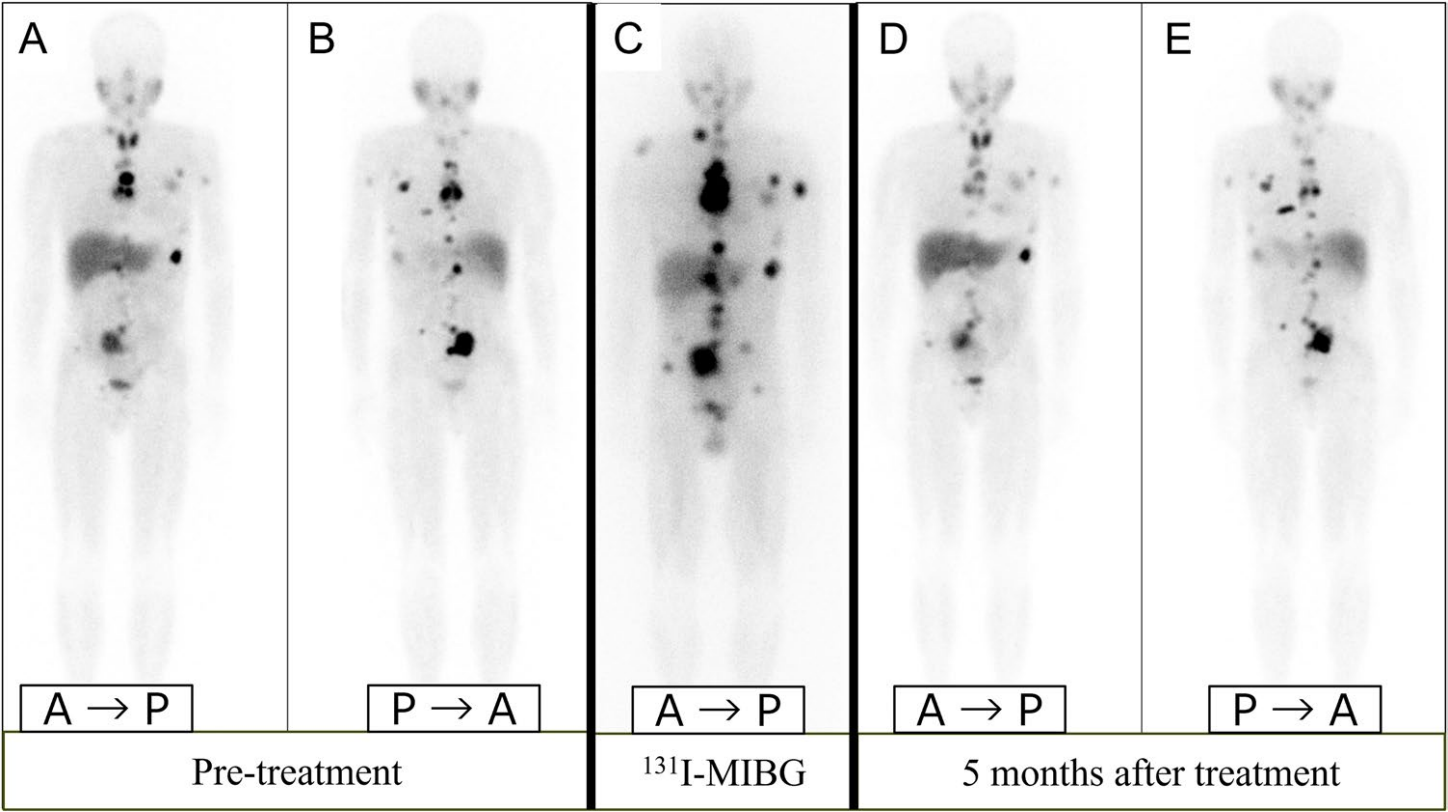
Pre- and post ^123^I-metaiodobenzylguanidine (^123^I-MIBG) therapy in malignant pheochromocytoma. A 60-year-old man with malignant pheochromocytoma. **A**, **B** Pre-treatment ^123^I-MIBG scintigraphy images in the anterior (**A**) and posterior (**B**) views show multiple areas of radiotracer accumulation, indicating widespread metastatic disease. **C** ^131^I-MIBG posttherapy scan showing increased radiotracer accumulation in the lesions. The posttherapy scan demonstrates more prominent uptake than the pre-treatment ^123^I-MIBG images. **D**, **E** ^123^I-MIBG scintigraphy images taken 5 months after treatment show a significant reduction in radiotracer uptake, suggesting a positive response to therapy. Urinary norepinephrine levels decreased from 1620 µg/day before treatment to 477 µg/day after the second treatment, further supporting the treatment efficacy

Functional imaging, such as ^123^I-MIBG scintigraphy, is crucial for identifying PPGLs and metastatic lesions and guiding treatment (Fig. 10, 11) [91, 92]. ^123^I-MIBG accumulates in catecholamine storage granules, so it can effectively visualize these tumors. Despite its widespread use, the sensitivity of ^123^I-MIBG for detecting PPGLs varies widely, ranging from 30 to 90% [7, 8, 93], with a specificity of about 94% [8]. However, the effectiveness of ^123^I-MIBG scintigraphy can be compromised, leading to false-negative results in certain scenarios, which include SDH-related PPGLs, especially those with SDHB mutations [94] in which the tumor biology affects ^123^I-MIBG uptake (Fig. 12). Other factors contributing to false negatives include tumors < 7 mm, cystic degeneration, necrosis, hemorrhage, and low VMAT1 expression in poorly differentiated PPGLs. Additionally, medications used to control hypertension or tachyarrhythmias in PPGL patients can influence the results [95]. In such cases, alternative imaging modalities, such as ^18^F-FDG PET/CT or ^68^ Ga-DOTATATE PET/CT, may offer more accurate detection and characterization, particularly for SDH-related tumors [95]. Although advances in CT and MRI have limited ^123^I-MIBG’s utility [96], it remains a practical option if more advanced imaging is unavailable. ^123^I-MIBG scintigraphy can be broadly performed in various regions to evaluate PPGLs, particularly for identifying metastatic lesions and determining treatment strategies (Fig. 13).

Regarding prognosis, all PPGLs are thought to have malignant potential, with a metastatic risk of 10–20% [84]. The primary treatment remains complete surgical excision, complemented by lifelong monitoring due to the risk of recurrence and metastasis. If the disease is metastatic or inoperable, adjuvant therapies, such as radiotherapy with ^131^I-MIBG, and targeted therapies are utilized [97, 98]. The efficacy of ^131^I-MIBG therapy in treating patients with advanced PPGLs varies significantly depending on several factors, including tumor burden and MIBG avidity. According to a multicenter phase 2 trial, high-dose ^131^I-MIBG therapy resulted in a partial response (PR) or stable disease (SD) in 92% of patients, with a median overall survival of approximately 37 months [99]. In a meta-analysis, the tumor response rates for conventional ^131^I-MIBG therapy were found to be 27% for PR and 52% for SD [100]. The variability in response highlights the importance of personalized treatment approaches, considering that ^131^I-MIBG therapy offers a viable option for controlling disease progression and managing symptoms in a significant proportion of patients with advanced PPGLs (Fig. 13). This comprehensive approach, which combines advanced imaging, genetic testing, and personalized clinical management, is essential for optimizing outcomes in patients with PPGLs.

## Neuroblastic tumors

### Neuroblastoma

Neuroblastoma is a highly aggressive pediatric malignancy originating from the adrenal medulla or sympathetic chain. It is the most common extracranial solid tumor in children, accounting for a significant percentage of childhood cancer deaths [101]. Most cases are diagnosed before 5 years of age, often presenting as large heterogeneous masses with necrosis, hemorrhage, and calcification, so imaging is crucial for diagnosis and staging [102, 103].

**Fig. 14:**
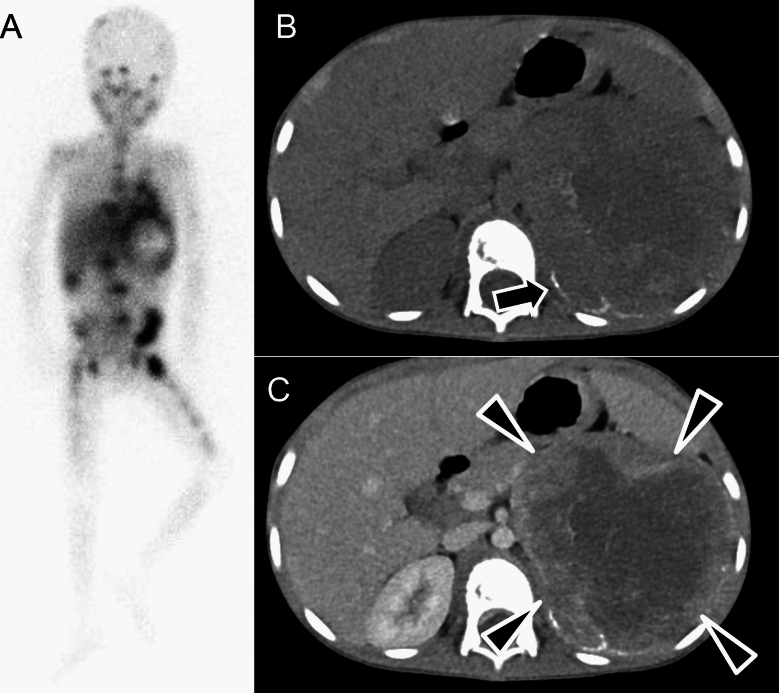
Imaging findings of the neuroblastoma. A 5-year-old male, diagnosed with Stage IV neuroblastoma, MYCN nonamplified. **A** ^123^I-metaiodobenzylguanidine scintigraphy showing radiotracer accumulation in the periphery of the left-retroperitoneal mass, with multiple bone metastases (calvarium, vertebrae, left ilium, right-sciatic bone, and bilateral femurs) and liver metastasis. **B** Unenhanced and enhanced computed tomography (CT) images showing a large left-retroperitoneal mass with calcification (arrows). **C** Enhanced CT showing peripheral enhancement (arrowhead) with extensive internal necrosis, characteristic of advanced neuroblastoma

Ultrasonography is typically the first imaging technique used, followed by CT or MRI for further characterization and staging. On ultrasonography, neuroblastomas appear as solid heterogeneous masses with calcifications in 30–90% of cases. Doppler ultrasonography can assess the vasculature as these tumors tend to encase or displace vessels [104]. On CT, neuroblastomas present as poorly circumscribed masses with heterogeneous attenuation due to necrosis and calcification, with the latter seen in 80–90% of cases [103–105]. They often encase structures, such as vessels or kidneys without infiltrating them, and bone involvement, including bone marrow metastases, is common (Fig. 14). MRI is useful for assessing tumor extent, particularly in cases with intraspinal involvement [102]. Neuroblastomas show heterogeneous signal intensity on T2WI, and contrast-enhanced MRI helps delineate tumor margins [104]. Wilms’ tumor (WT) is a critical differential diagnosis for adrenal neuroblastoma. Unlike neuroblastoma, which tends to displace the kidney inferiorly and cross the midline, WT affects the kidney and often extends into the renal vein or IVC and forms a tumor thrombus [106]. Both neuroblastoma and WT exhibit lower ADC values, but the presence of calcifications are characteristic of neuroblastomas, which can help distinguish them from WT [107].

^123^I-MIBG scintigraphy is the gold standard for detecting primary tumors and metastases, showing uptake in 67–100% of neuroblastomas, although poorly differentiated tumors may yield false negatives [108]. ^18^F-FDG-PET/CT is useful when ^123^I-MIBG is negative. Bone metastases are the most common form of neuroblastoma spread, and their presence is strongly associated with prognosis, so their accurate evaluation is essential [109]. Both modalities are also use for monitoring treatment response and detecting residual or recurrent disease because MRI abnormalities often persist after treatment and are not ideal for assessing therapeutic efficacy [102, 110]. Staging and risk classification rely on both imaging and clinical factors, with the International Neuroblastoma Risk Group Staging System [111] or revised classification system [112] incorporating imaging-defined risk factors, such as vascular encasement and intraspinal extension to guide treatment.

Radiologists are essential in the management of neuroblastoma from diagnosis to follow-up by helping with tumor staging, treatment planning, and monitoring for metastasis, recurrence, or treatment complications. A multidisciplinary approach is often necessary for comprehensive patient care.

### Ganglioneuroma

Ganglioneuromas are rare benign tumors arising from neural crest cells, and primarily composed of well-differentiated ganglion cells, Schwann cells, and fibrous tissue. These tumors are the mature benign counterpart of neuroblastomas but lack the immature neuroblasts found in more malignant variants, such as ganglioneuroblastomas [113], which explains their favorable prognosis and low recurrence rate, even after incomplete resection. Ganglioneuromas typically occur in the sympathetic nervous system, particularly in the posterior mediastinum and retroperitoneum [114], but they also can be found in the adrenal glands [114], cervical region [115], and spinal canal [116]. The median age at diagnosis was reported as 7.5 years, with a slight female predominance [115]. These tumors are usually asymptomatic and often found incidentally, although large tumors may compress nearby structures and cause symptoms requiring surgery [114].

**Fig. 15:**
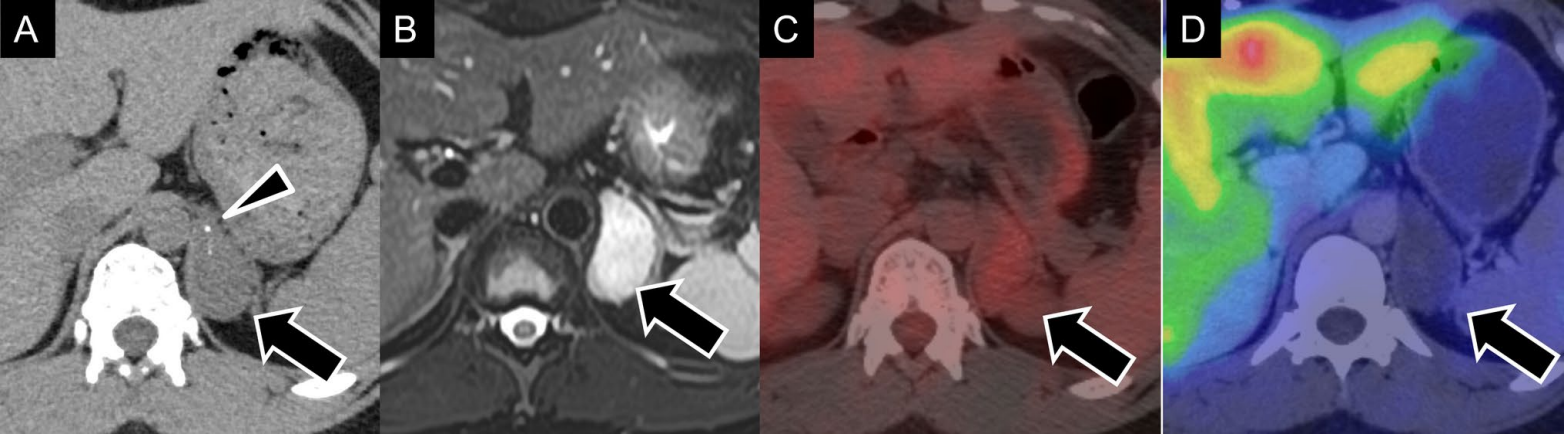
Imaging findings of the ganglioneuroma. A 31-year-old female with an incidentaloma. **A** Unenhanced computed tomography (CT) showing a well-circumscribed mass in the left = adrenal gland (arrow) with fine speckled calcifications (arrowhead). **B** T2-weighted magnetic resonance imaging showing a high signal intensity of the mass (arrow). **C** Fusion single-photon-emission computed tomography/computed tomography images showing mild ^123^I-metaiodobenzylguanidine uptake in the lesion (arrow), which can occasionally be seen in ganglioneuroma, although negative scans are more typical. **D** Fusion positron-emission tomography/computed tomography images showing no significant ^1^⁸F-fluorodeoxyglucose (^1^⁸FDG) uptake within the tumor (arrow), consistent with ganglioneuroma. Pathology confirmed the diagnosis of ganglioneuroma

On CT, ganglioneuromas appear as oval or lobulated, well-defined, encapsulated, hypo-attenuating masses [117] with calcification in approximately 20% of cases [103], usually fine and punctate, although coarse calcification may occasionally occur [118]. On MRI, ganglioneuromas show heterogeneous intermediate-to-high intensity on T2WI, with a characteristic “whorl sign” or “stripe sign,” representing the intersection of Schwann cells and collagen fibers within hyperintense areas (Fig. 15) [117, 119]. The mucous-like matrix leads to delayed enhancement on post-contrast imaging, and larger tumors may exhibit cystic degeneration. DWI typically shows hyperintensity, which helps differentiate ganglioneuroma from cystic lesions. Large ganglioneuromas can occasionally mimic malignant tumors, but they can usually be distinguished from neuroblastomas based on imaging findings, as neuroblastomas tend to exhibit more amorphous calcifications and higher uptake of ^123^I-MIBG and ^1^⁸FDG [120, 121].

## Other tumors: commonly encountered diseases or diseases that require differentiation by diagnostic imaging

### Myelolipoma

**Fig. 16:**
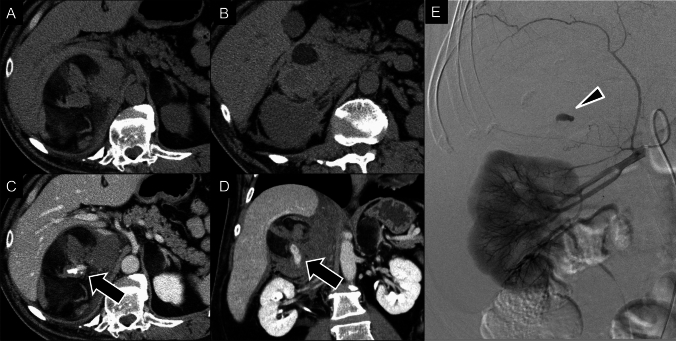
Retroperitoneal hemorrhage from adrenal myelolipoma. A 38-year-old male presented with right-upper quadrant pain. **A**, **B** Unenhanced computed tomography (CT) images showing a right-adrenal mass containing macroscopic fat, suspected to be an adrenal myelolipoma or cortical adenoma with myelolipomatous degeneration. The tumor had a growth rate of 8 mm/year prior to this imaging. **C**, **D** Enhanced CT showing contrast media extravasation within the mass, confirming active bleeding (arrows). **E** Right-renal angiography showing active extravasation from the right-inferior adrenal artery (arrowhead), which was successfully treated with glue embolization

Myelolipomas are benign tumors consisting of mature adipose tissue and hematopoietic cells, accounting for 3.3–6.5% of all primary adrenal tumors [122, 123]. They are usually asymptomatic and incidentally discovered on imaging, but large tumors may cause nonspecific abdominal pain due to compression, and those > 10-cm risk rupture or hemorrhage [124], potentially requiring surgery (Fig. 16).

The pathogenesis of myelolipomas is unclear, but theories involve progenitor-cell interactions, metaplastic changes due to stress or inflammation, and hormonal factors, such as ACTH overexpression [125, 126]. Approximately 10% of cases are associated with CAH, often presenting bilaterally [126].

On imaging, myelolipoma appeared as well-circumscribed, heterogeneous masses. US shows hypoechoic lesions, and CT reveals mixed fat and soft tissue with low attenuation due to macroscopic fat [124]. Calcifications and contrast enhancement of soft-tissue components can occasionally be observed [124]. MRI shows hyperintense fatty components on T1WI with signal loss on fat-suppressed sequences [127, 128]. Hemorrhage may be visible on CT or MRI, depending on the blood degradation products [129, 130].

In most cases, encapsulated lesions with fat and soft tissue are confidently diagnosed as myelolipomas. However, extra-adrenal myelolipomas account for 10–15% of cases, and those found in the retroperitoneum and other areas present diagnostic challenges. The differential diagnosis includes liposarcomas, angiolipomas, neurogenic tumors, and extramedullary hematopoiesis [124, 131–133]. Unlike liposarcomas, myelolipomas lack irregular margins and heterogeneous enhancement, which aids in differentiation. Importantly, ^99m^Tc-sulfur colloid scintigraphy has proven useful in distinguishing myelolipomas from other fat-containing masses, particularly extra-adrenal lesions [134].

In summary, adrenal and extra-adrenal myelolipomas are stable, nonmalignant tumors that are generally managed by monitoring with appropriate follow-up if needed. However, tumors > 8 cm or symptomatic cases may require surgical excision due to the risk of hemorrhage or rupture, and differentiating them from other adrenal or retroperitoneal masses is crucial for the appropriate management.

### Lymphoma

Adrenal lymphoma, a rare extranodal non-Hodgkin lymphoma subtype that comprises < 1% of cases, primarily presents as diffuse large B-cell lymphoma [135]. It can occur as primary adrenal lymphoma (PAL) or as secondary involvement in systemic lymphoma. PAL typically presents bilaterally and with abdominal pain, followed by B symptoms, such as fever, weight loss, or night sweating, and less commonly, adrenal insufficiency [135, 136]. Secondary adrenal involvement is more common and usually associated with widespread systemic lymphoma.

**Fig. 17:**
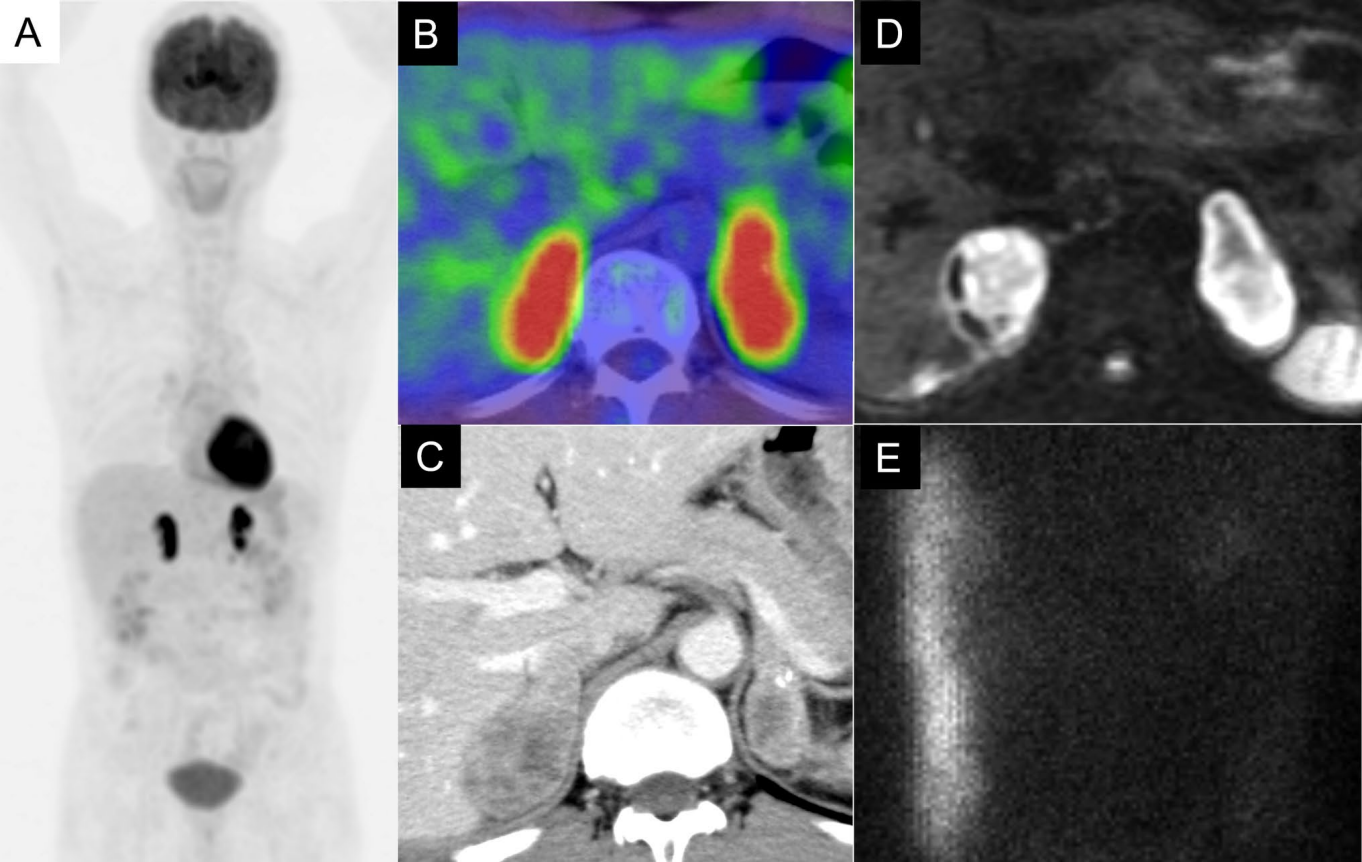
Imaging features of adrenal lymphoma. A 63-year-old man with weight loss who was pathologically confirmed to have primary adrenal lymphoma. **A** ^1^⁸F-fluorodeoxyglucose (^1^⁸FDG) positron-emission tomography (PET) maximum-intensity projection image showing intense radiotracer uptake in the bilateral adrenal masses. **B** Fusion PET/computed tomography (CT) image highlights ^1^⁸FDG uptake in the adrenal lesions. **C** Contrast-enhanced CT revealing bilateral bulky adrenal masses with heterogeneous enhancement. **D** Diffusion-weighted image on magnetic resonance imaging showing the high signal intensity of the masses. **E** Adrenocortical scintigraphy is negative for abnormal uptake, which is commonly seen in adrenal lymphoma.

Adrenal lymphoma typically presents as a large mass, with an average tumor size of 8–10 cm, appearing as a well-defined soft-tissue density mass on CT and showing diffusion restriction on MRI (Fig. 17) [137, 138]. Lesions show mild progressive contrast enhancement without marked necrosis or hemorrhage. Immune deficiency/dysregulation-associated lymphoid proliferative disorders (LPDs), such as methotrexate-associated LPD, are often Epstein–Barr virus related [139], and necrosis and hemorrhage are more common, complicating differentiation from infections [140]. ^18^F-FDG PET/CT is useful for diagnosing lymphoma and is superior to CT in visualizing extra-adrenal lesions [141]. However, bilateral high ^18^F-FDG uptake is not specific and can also be seen in other conditions, such as infections (e.g., tuberculosis) or vagal reflexes. The prognosis is generally poor, with chemotherapy, often using the R-CHOP regimen, as the primary treatment [142].

### Metastasis

Metastases are the most common adrenal malignant lesions, typically discovered during the staging or follow-up of malignancies. Primary cancers that frequently metastasize to the adrenal glands include lung, breast, melanoma, renal, gastrointestinal, and pancreatic cancers [143, 144].

**Fig. 18:**
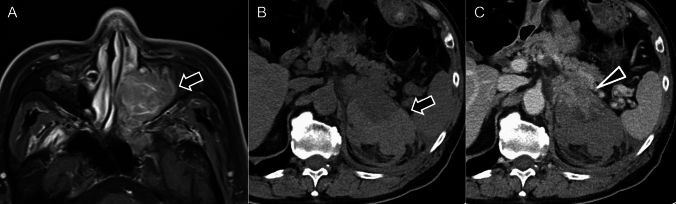
Imaging of adrenal metastasis. Patient with angiosarcoma of the maxillary sinus. **A** Magnetic resonance imaging of the maxillary sinus showing a mass occupying the left-maxillary sinus with heterogeneous enhancement on gadolinium-enhanced T1-weighted imaging (arrow). **B** Unenhanced CT showing a high-density area dorsally to the left-adrenal mass, suggesting retroperitoneal hemorrhage (arrow). **C** Enhanced computed tomography showing irregular tumor enhancement in the left-adrenal mass (arrow), consistent with metastasis. Adrenal metastasis can occasionally be complicated by hemorrhage

Metastases appear bilaterally in 32–73% of cases and are often incidentally discovered on imaging [143]. On CT and MRI, adrenal metastases appear as large well-defined masses with heterogeneous enhancement and areas of necrosis or hemorrhage (Fig. 18). They often show delayed contrast washout relative to that of adenomas, which show rapid washout [3]. ^18^F-FDG PET/CT helps differentiate adrenal metastases from benign lesions [145] since metastases typically show significantly higher ^18^F-FDG uptake [143]. However, false positives can occur in benign or inflammatory lesions, such as adrenalitis caused by ICIs, which may also show high ^18^F-FDG uptake [14, 143–147]. The combination of PET with CT or MRI improves differentiation. If biopsy is required, ruling out PPGLs is essential to avoid hypertensive complications.

### Adrenal hemorrhage and adrenal infarction

Adrenal hemorrhage and infarction are rare but potentially life-threatening conditions associated with various causes. Adrenal hemorrhage is caused by trauma, anticoagulation, coagulopathies, infections, or severe stress [148]. Hemorrhage following sepsis, known as Waterhouse–Friderichsen syndrome, commonly causes bilateral adrenal involvement [149]. Coagulopathy-related causes include disseminated intravascular coagulation, antiphospholipid syndrome (APS), heparin-induced thrombocytopenia, and adrenal tumors. In neonates, the causes include birth trauma, hypoxia, hemorrhagic disorders, and maternal diabetes [148]. Adrenal-vein thrombosis is another recognized cause. Symptoms range from nonspecific abdominal or back pain to adrenal crisis with bilateral involvement. On CT, the acute hemorrhage appears as a round or oval high-attenuation mass, which decreases over time. MRI is particularly useful for estimating the timing of the hemorrhage, with subacute hemorrhages appearing hyperintense on T1WI and T2WI. Chronic hemorrhages may show calcification and can appear as pseudocysts on imaging [150].

**Fig. 19:**
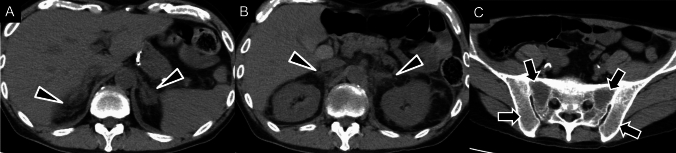
Adrenal Infarction in a patient with myelodysplastic syndrome. A male in his 50 s presenting with acute onset of abdominal pain. **A**, **B** Unenhanced computed tomography (CT) images showing swelling of the bilateral adrenal glands and increased surrounding fat density (arrowheads), which are consistent with an adrenal infarction. **C** CT image revealing elevated density in the pelvis and bilateral iliac bones (arrows), probably associated with atypical cells from myelodysplastic syndrome.

Adrenal infarction, although rare, can result from certain conditions, such as APS, myelodysplastic syndrome (Fig. 19), and TAFRO syndrome [151]. APS, which is often associated with adrenal infarction, leads to venous thrombosis and subsequent hemorrhagic infarction. CT typically shows adrenal enlargement with fat stranding and a lack of enhancement, characteristic of infarction. In TAFRO syndrome, adrenal infarction can be accompanied by systemic symptoms, such as pleural effusion and lymphadenopathy, and bilateral adrenal enlargement is often observed on imaging [151]. Severe acute respiratory syndrome coronavirus 2 (SARS-CoV-2), or coronavirus 2019 (COVID-19) infection can also cause adrenal infarction due to thromboembolic complications, worsening prognosis in critically ill patients [152]. Both conditions require prompt diagnosis and management, especially in bilateral cases, to prevent adrenal insufficiency. Treatment is often conservative, with adrenal replacement therapy needed in cases of significant insufficiency. Interventional procedures, such as embolization, may be required for ongoing hemorrhage or infarction unresponsive to medical management.

### Infectious disease

**Fig. 20:**
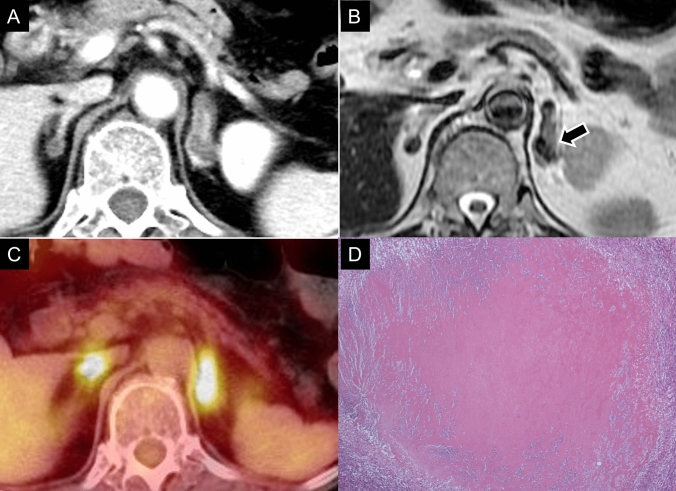
Adrenal tuberculosis. A 74-year-old female presented with an incidental adrenal mass. **A** Enhanced computed tomography (CT) showing bilateral adrenal masses with low attenuation. **B** T2-weighted magnetic resonance imaging showing low signal intensity in the lesions (arrow). **C** ^1^⁸F-fluorodeoxyglucose positron-emission tomography showing high uptake in the bilateral adrenal masses. **D** Pathological analysis confirmed caseating necrosis, leading to the diagnosis of adrenal tuberculosis

Infections involving the adrenal glands, although uncommon, can cause significant adrenal pathology. Tuberculosis remains one of the leading infectious causes, particularly in regions where it is endemic. Adrenal involvement typically occurs bilaterally and may lead to adrenal insufficiency [147]. On CT, tuberculosis often presents as bilateral enlargement with low attenuation [153]. Calcification or atrophy may occur in the chronic stage [147]. MRI may reveal mixed signal intensities, with central hypointensity on T2WI and peripheral rim enhancement (Fig. 20) [154]. Generally, caseous necrosis can sometimes show high signal intensity on T1WI [155].

Fungal infections, especially disseminated histoplasmosis in immunocompromised patients, occasionally involve the adrenal glands, presenting as bilateral adrenal masses with peripheral enhancement on imaging. CT may show enlarged glands with low attenuation, and MRI often reveals heterogeneous signal intensity.

Viral infections such as cytomegalovirus occur in neonates or immunocompromised individuals, causing adrenal enlargement and sometimes insufficiency [156]. Although imaging findings for viral infections are nonspecific, adrenal involvement may be incidentally detected by ^18^F-FDG PET/CT during evaluations for other conditions in immunocompetent individuals [157].

Infections, although rare causes of adrenal disease, should be considered in immunocompromised patients or in areas with endemic infectious diseases. Imaging has a key role in identifying these conditions, but clinical correlation and histopathological confirmation, when necessary, remain essential for accurate diagnosis.

## Conclusion

Adrenal imaging requires a multifaceted approach that uses CT, MRI, and nuclear medicine to accurately diagnose and manage a wide spectrum of adrenal disorders. This review highlighted the key imaging features of various conditions and the complementary roles of different modalities, providing a valuable resource for radiologists and trainees in clinical practice. To gain a comprehensive understanding of adrenal imaging, it is essential to first have a solid grasp of the anatomy and physiology of the adrenal glands. A thorough knowledge of adrenal cortical adenomas, endocrine abnormalities, PPGL, and neuroblastomas through CT, MRI, and nuclear medicine studies is critical. Staying updated on recent advancements including evolving terminology, such as adrenal cortical disease, genetic correlations, hereditary tumor syndromes, and the use of nuclear medicine therapies, is equally important. Finally, understanding the differential diagnoses, key conditions, and systemic diseases involving the adrenal glands is fundamental to ensuring accurate imaging interpretation and effective diagnosis of adrenal disorders.
